# MiR-21 Is Required for the Epithelial–Mesenchymal Transition in MDA-MB-231 Breast Cancer Cells

**DOI:** 10.3390/ijms22041557

**Published:** 2021-02-04

**Authors:** Elif Damla Arisan, Ozge Rencuzogullari, Clara Cieza-Borrella, Francesc Miralles Arenas, Miriam Dwek, Sigrun Lange, Pinar Uysal-Onganer

**Affiliations:** 1 Institute of Biotechnology, Gebze Technical University, Gebze, 41400 Kocaeli, Turkey; damlaarisan@gmail.com; 2 Department of Molecular Biology and Genetics, Atakoy Campus, Istanbul Kultur University, 34156 Istanbul, Turkey; ozgeberrak@gmail.com; 3 Centre for Biomedical Education/Cell Biology and Genetics Research Centre, St. George’s, University of London, Cranmer Terrace, London SW17 0RE, UK; ccieza-b@sgul.ac.uk (C.C.-B.); fmiralle@sgul.ac.uk (F.M.A.); 4 Cancer Research Group, School of Life Sciences, College of Liberal Arts and Sciences, University of Westminster, 115 New Cavendish Street, London W1W 6UW, UK; m.dwek@westminster.ac.uk; 5 Tissue Architecture and Regeneration Research Group, School of Life Sciences, University of Westminster, London W1W 6UW, UK; s.lange@westminster.ac.uk

**Keywords:** breast cancer, miR-21, Wnt-11, EMT

## Abstract

Breast cancer (BCa) is one of the leading health problems among women. Although significant achievements have led to advanced therapeutic success with targeted therapy options, more efforts are required for different subtypes of tumors and according to genomic, transcriptomic, and proteomic alterations. This study underlines the role of microRNA-21 (miR-21) in metastatic MDA-MB-231 breast cancer cells. Following the knockout of miR-21 from MDA-MB-231 cells, which have the highest miR-21 expression levels compared to MCF-7 and SK-BR-3 BCa cells, a decrease in epithelial-mesenchymal transition (EMT) via downregulation of mesenchymal markers was observed. Wnt-11 was a critical target for miR-21, and the Wnt-11 related signaling axis was altered in the stable miR-21 knockout cells. miR-21 expression was associated with a significant increase in mesenchymal markers in MDA-MB-231 BCa cells. Furthermore, the release of extracellular vesicles (EVs) was significantly reduced in the miR-21 KO cells, alongside a significant reduction in relative miR-21 export in EV cargo, compared with control cells. We conclude that miR-21 is a leading factor involved in mesenchymal transition in MDA-MB-231 BCa. Future therapeutic strategies could focus on its role in the treatment of metastatic breast cancer.

## 1. Introduction

Breast cancer (BCa) is one of the most frequent malignancies and the second most common cause of cancer-associated mortality worldwide [1,2]. Treatment includes surgical intervention, radiotherapy (RT), systemic therapy with cytotoxic chemotherapy, hormone therapy, biological therapy, or a combination of all these therapies [3]. After breast-conserving surgery, the tumor bed represents the region with the highest recurrence (≤90%) [4]. In recent years, regular BCa screening measurements helped diagnose the tumors in more curable stages. However, BCa remains a significant clinical problem because of the poor prognosis of some of the subtypes of the disease, for which there are limited treatment options and frequent development of resistance to chemotherapy. Triple-negative breast cancer (TNBC) is characterized by a lack of estrogen and progesterone receptor expression and negative amplification of the human epidermal growth factor gene. Approximately 15% of all breast cancers are TNBC, and a poor prognosis fails to identify the disease early in its etiology; thus, TNBC is capable of metastasis prior to diagnosis [5,6]. The current therapies are mostly insufficient to eradicate metastatic cells; therefore, identifying novel mechanisms underlying TNBC development and metastasis and elucidating new treatment targets is critical.

microRNAs (miRs) are small RNA molecules (20–24 nucleotides in lengths) involved in the negative regulation of gene expression at the post-transcriptional level [7]. It has been shown that miRs are involved in cell proliferation, apoptosis, control of development and cellular responses to stress [8]. Dysfunction of miRs has been associated with several diseases such as cardiovascular [9], autoimmune [10] and many different types of malignancies, including BCa [11,12,13,14,15,16]. miR-21 has been reported to be upregulated and to promote metastasis in several tumor types [17,18,19,20]. Our previous studies showed that miR-21 is involved in cancer cell invasion and differentiation [21,22,23,24].

The epithelial-mesenchymal transition (EMT) is a process whereby epithelial cells lose their cell polarity and cell–cell adhesion, key steps in the metastatic process [25,26]. Several miRs have been reported to be associated with the progression of TNBC, specifically regulating EMT, stem cell-like differentiation, invasiveness, and migration [27]. During EMT, E-cadherin, a protein required for normal epithelial cell maintenance, diminishes while transcriptional factors such as Zeb1, Zeb2, Snail1, Slug are positively expressed and, via binding E-cadherin promoter, actively repress its transcription [28,29,30,31]. miR-21 has been shown to be involved in EMT; however, the molecular mechanism underlying the regulation of EMT processes by miR-21 in BCa is not precisely understood. It has demonstrated that TGFβ activation modules miR-21 expression in cancer cells, which governs EMT. Like other miRs, miR-21 regulates the expression of many genes such as PTEN, HIF1α, and leucine zipper transcription factor-like 1 (LZTFL1) in BCa [32,33,34]. Moreover, upregulated expression of miR-21 was reported in the tumor stroma, which was associated with poor prognosis in TNBC [35]. We have previously reported that miR-21 modulates Wnt signaling. Wnt-11 is one of the noncanonical Wnt family members found to be activated during carcinogenesis, leading to a poor prognosis. Moreover, Wnt-11 leads to neuroendocrine differentiation of different prostate cancer cell lines [22,36] and orchestrates many proteins, including PKC, JNK, NF-κB, Rho, PKA, PI3K, and crosstalk between Wnt-11 and TGF-β was demonstrated [37]. Interestingly, it was reported that miR-21 is one of few miRNAs whose processing is regulated by TGF-β [38]. Wnt-11 levels are associated with cancer stemness, and it is recognized that breast cancer stem cells contributed to tumorigenesis, metastasis and chemotherapeutic resistance [39]. Stemness markers, such as ALDH1, modulate the early differentiation of stem cells and have been shown to be prognostic markers in lymph node-positive breast cancer [40]. GATA-2, a transcription factor and a stemness biomarker, was negatively correlated with PTEN expression in BCa [41]. Since PTEN is a direct target of miR-21, miR-21 expression levels via AKT signaling in cancer affect the survival mechanism. Several studies reported that miR-21 regulates the cancer stem cell phenotype and EMT by targeting PTEN and AKT pathways and knocking down miR-21 reverses EMT and cancer stem cell phenotypes [42].

Extracellular vesicles (EVs) have been shown to mediate pro-oncogenic changes. These lipid bilayer-enclosed structures, 30–1000 nm in diameter, are released from cells and act as mediators for intra/inter-tumor communication through horizontal transfer of functional proteins and nucleic acids (mRNA, miRNA, lncRNA, sncRNA) [43,44,45,46]. EVs have great potential as diagnostic and prognostic biomarkers in various pathologies and have received considerable attention in a range of cancers, including BCa [47,48]. The release of EVs by cancer cells leads to both intercellular communication within a tumor and influences the surrounding microenvironment promoting tumor growth, angiogenesis, metabolism, and invasion [47]. Therefore, EV modulating strategies are of considerable interest, and a range of studies has identified that various agents inhibiting or modulating EV release from cancer cells can contribute to decreasing in chemoresistance as well as reducing tumor growth in vivo [49,50,51,52,53,54,55,56,57,58].

Here, we investigated the mechanism of miR-21 in regulating cellular differentiation and invasion by generating a miR-21 knockout BCa cell line. We demonstrated a novel mechanism by which miR-21 regulates EMT highlighting the importance of miR-21 expression in BCa, and furthermore found that EV release is significantly reduced in miR-21 KO cells.

## 2. Results

Four sets of results are presented. First, we show the potential role of miR-21 using The Cancer Genome Atlas Breast Invasive Carcinoma (TCGA-BRCA) database and the miR-21 expression levels reported for BCa cell lines; then, we adopted the MDA-MB-231 cells as a model to generate miR-21 stable knockout (KO) cells. Second, we show the results of inhibition of miR-21 expression and decrease in the cellular invasion, as well as affecting EV release profiles. Third, we demonstrate that miR-21 modulates EMT and Wnt signaling pathways. Fourth, we reveal that by adding miR-21 back to the KO cells, we reverse the central cellular behavior of EMT. Overall, these results suggest that miR-21 plays a significant role in the pathophysiology of TNBC.

### 2.1. miR-21 Decreased Disease-Specific and Overall Survival Rates in Breast Cancer In Vivo and In Vitro

**Figure 1:**
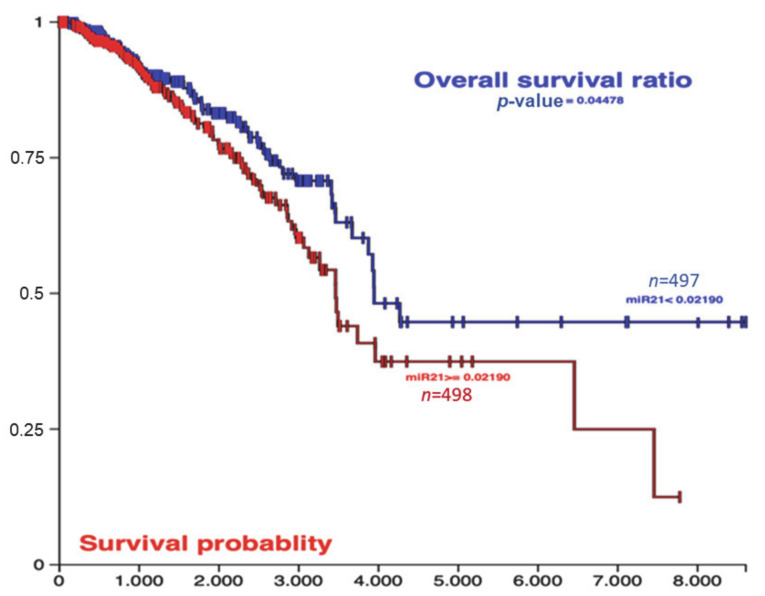
TCGA Breast Cancer (BRCA) database results were analyzed by the UCSC Xena tool [59] according to the expression profile of miR-21, low or high. Kaplan–Meier plots were used for the overall survival ratio for miR-21 low and high expression profile patients (995 patients in total). x-axis survival time cutoff, y-axis survival probability.

According to the breast cancer BRCA database in the TCGA of 995 breast cancer patients, the high expression profile of miR-21 was significantly correlated with overall survival (Figure 1).

**Figure 2:**
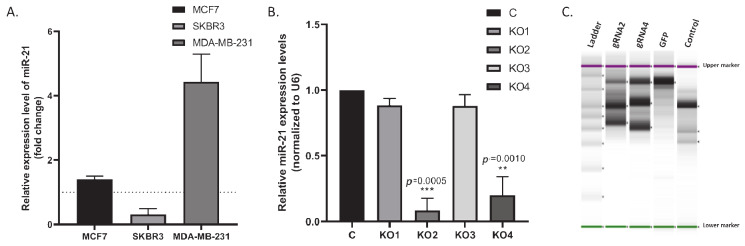
(**A**) miR-21 expression levels were analyzed by qRT–PCR in MCF-7, SKB3, and MDA-MB-231 cells. The column graphic represents the average of three replicates of RNA isolated from each cell line. Data normalized according to RNU6 expression level by fold analysis (*n* = 3; *p* < 0.005). (**B**) miR-21 knockout MDA-MB-231 cell colonies confirmed by qRT–PCR assay. The column graphic represents the average of three replicates of RNA isolated from each cell line. miR-21 expression levels of miR-21 knockout clone 2 (KO2) and miR-21 knockout clone 4 (KO4) cell colonies were downregulated significantly compared to untreated control MDA-MB-231 cells (*n* = 3; *p* = 0.005 and 0.001, respectively). ** *p* = 0.0010, *** *p* = 0.0005. (**C**) genomic cleavage Assay TapeStation results indicating CRISPR/Cas9 efficiencies of 83.11% (gRNA2) and 75.37% (gRNA4) in comparison with GFP control (0%) and assay commercial’s control (0%). Fragments size: 25 bp lower marker, 1500 bp upper marker. ≈860 bp non-cleaved product, ≈540 bp cleavage product 1 and ≈320 cleavage product 2.

Starting from this point, the miR-21 expression level of MCF-7, SKBR3, and MDA-MB-231 BCa cells, was assessed, MDA-MB-231 cells expressed the highest miR-21 levels compared to MCF-7 and SKBR3 cells (4-fold; *n* = 3; *p* < 0.005; Figure 2A). MDA-MB-231 cells were adopted as a model system for further analysis and were transduced with four different miR-21 gRNAs and a control vector. Transduced cells were selected using puromycin (1–10 μg/mL). miR-21 expression analysis showed that miR-21 expression was significantly reduced in knockout (KO) clones 2 and 4 compared to vector-alone (50- and 10-fold; *n* = 3; *p* = 0.005 and 0.001, respectively; Figure 2B). The cleavage assay confirmed >60% transfection efficiency (Figure 2C). Electropherogram results also confirmed the deletion efficiency of the selected clones (Figure S1).

**Figure 3:**
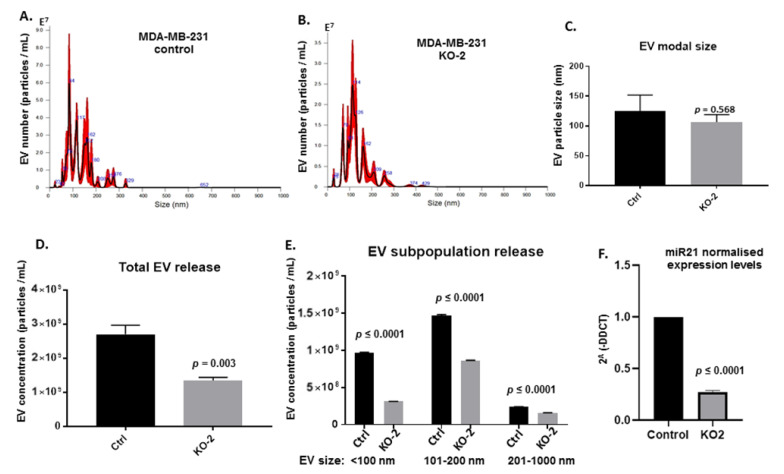
Extracellular vesicle (EV) analysis of miR-21 knockout MDA-MB-231 cells. (**A**) Representative nanoparticle tracking analysis (NTA) profile of extracellular vesicles (EVs) derived from control MDA-MB-231 cells. (**B**) Representative NTA profile of EVs derived from miR-21 knockout (KO-2) MDA-MB-231 cells. (**C**) EV modal size for EV profiles from control versus miR-21 (KO-2) MDA-MB-231 cells. (**D**) Total EV release is significantly reduced in the miR-21 KO cells. (**E**) Cellular release of all three EV subpopulations is significantly reduced in the miR-21 KO, compared with control MDA-MB-231 cells. (**F**) EV cargo assessed for miR-21 shows a significant reduction in miR-21 content in EVs derived from the miR-21 KO (70%), compared with EV miR-21 cargo from control MDA-MB-231 cells. Exact *p*-values are indicated (*n* = 3).

Due to the importance of EVs in cellular communication and cancer progression, EV release profiles and EV cargo were assessed in MDA-MB-231 cells, comparing KO and wt clones. Figure 3 shows representative NTA profiles for the EV size distribution profiles of wt control (Figure 3A) and clone 2 (KO2) (Figure 3B) clones, no significant effect on EV size distribution, including modal size, was observed (Figure 3C). On the other hand, significant changes were observed in the total amount of EVs released, with a 45% reduction in the KO2 clone compared with the wt control (*p* = 0.003; Figure 3D). When assessing differences in EV release of different EV subpopulations, a significant reduction in EV release was observed for the KO2 clone compared with wt, for small EVs (>100 nm; 60%, *p* ≤ 0.001), medium-sized EVs (101–200 nm; 45% reduction, *p* ≤ 0.001) and large EVs (201–1000 nm, 40% reduction, *p* ≤ 0.001). Furthermore, when assessing the amount of EV miR-21 cargo in KO2 versus in wt control EVs, a significant reduction (70%, *p* ≤ 0.001) was observed for miR-21 cargo (normalized with U6) in the KO2 cell-derived EVs (Figure 3F).

### 2.2. miR-21 Downregulation Decreased the Colony Size in MDA-MB-231 Cells via Increasing Survival-Related Mechanisms

**Figure 4:**
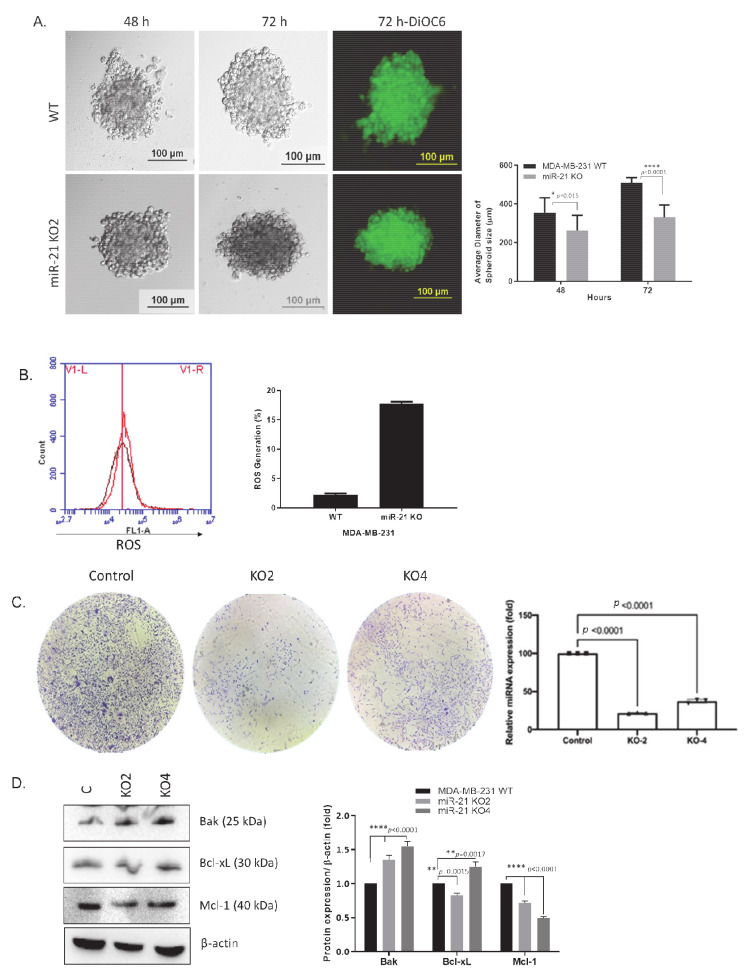
The silencing of miR-21 suppressed the growth of MDA-MB-231 breast cancer cells. (**A**) Hanging drop assay was adopted to understand the effect of miR-21 deletion on 3D spheroid growth of MDA-MB-231 wt and miR-21 KO2 MDA-MB-231 cells. The spheroid size was observed at 48 and 72 h. DiOC6 staining was performed to observe the viable cells in the spheroid structure of miR-21 KO2 and wt MDA-MB-231 BCa cells at 72 h. The scale bar is 100 µm. The average diameter of spheroids size was determined using at least ten spheroids. * *p* = 0.015, **** *p* < 0.0001 (**B**) DCFH-DA staining was used for the determination of the ROS levels following the deletion of miR-21 in MDA-MB-231 cells. Following 15 min incubation of cells with DCFH-DA, cells were harvested and analyzed by using flow cytometer FL1 channel for 1 × 10_4_ events (**C**). Colony formation assay for MDA-MB-231 wt and miR-21 KO cells. The colonies were observed with crystal violet staining of cells following 14 days. (**D**) The expression levels of pro-and antiapoptotic proteins were determined by Western blotting. The densitometry graph represents protein expression levels of band intensities obtained from at least three independent experiments. The relative band intensities of each sample were calculated by normalizing against β-actin. ** *p* < 0.002, **** *p* < 0.0001.

To investigate the biological function of miR-21, a spheroid formation assay was used following miR-21 inhibition. Spheroid formation of wt and miR-21 KO cells were assessed over 72 h. A significant reduction in spheroid formation in KO clones was observed when compared to wt (*n* = 3; *p* = 0.015 for 48 h and *p* < 0.001 for 72 h; Figure 4A). Reactive oxygen species (ROS) generation was used to evaluate the cellular stress level following miR-21 knockout and was observed to be increased in KO cells. (Figure 4B, Supplemental Figure S2). Similarly, the miR-21 KO cells exhibited a decrease in clonogenicity compared to wt cells after 14 days (Figure 4C). To assess whether the apparent reduction in proliferation was due to increased apoptosis, the levels of proteins associated with apoptosis (Bak, Bcl-xL and Mcl-1) were assessed. Interestingly, the protein levels for both Bak and Bcl-xL were increased in the KO clones, whereas the apoptotic regulator Mcl-1 protein level was significantly reduced (*n* = 3; Figure 4D).

### 2.3. miR-21 Promotes EMT

**Figure 5:**
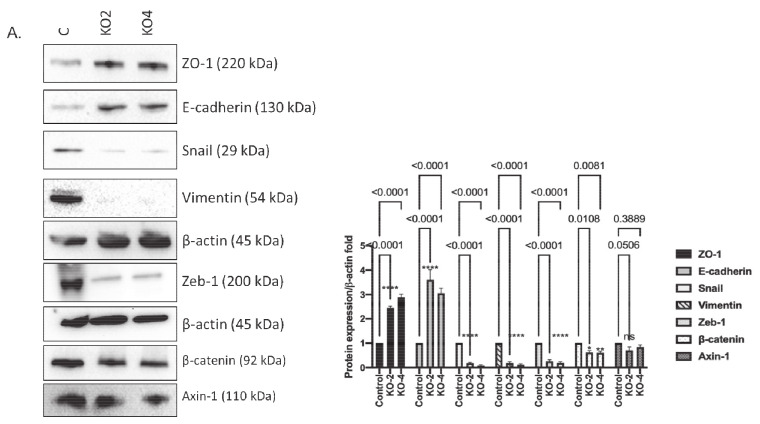

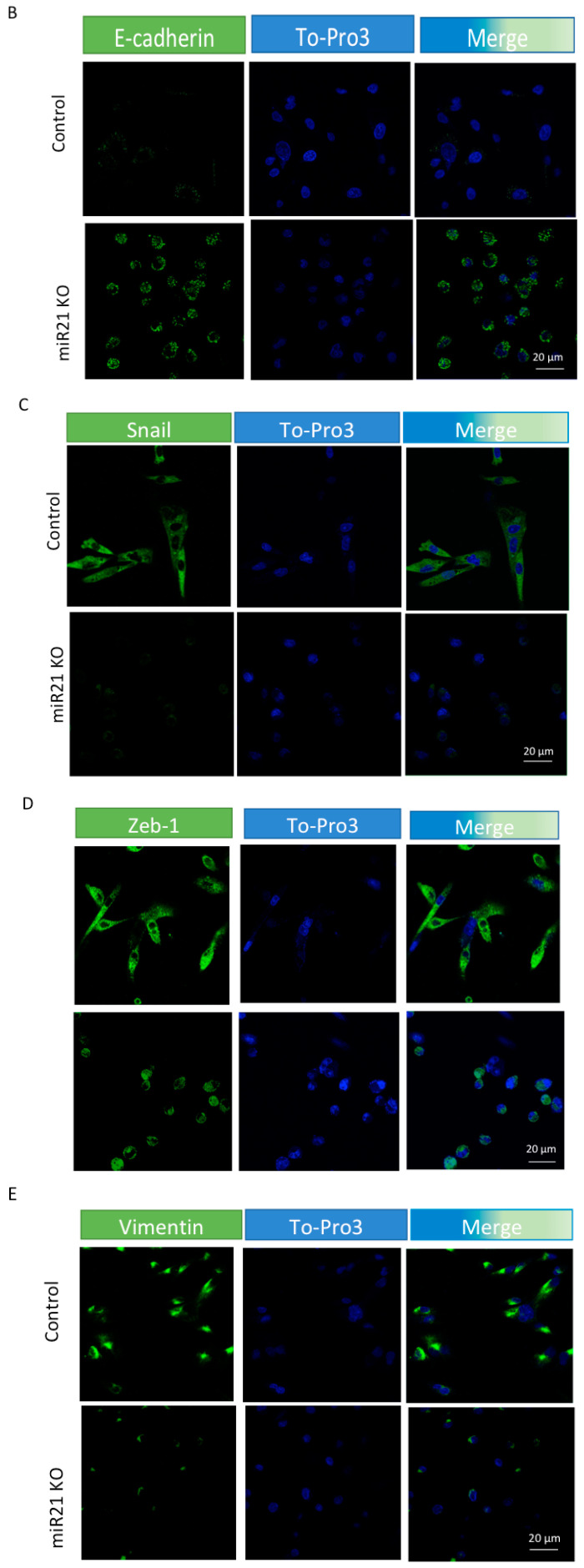

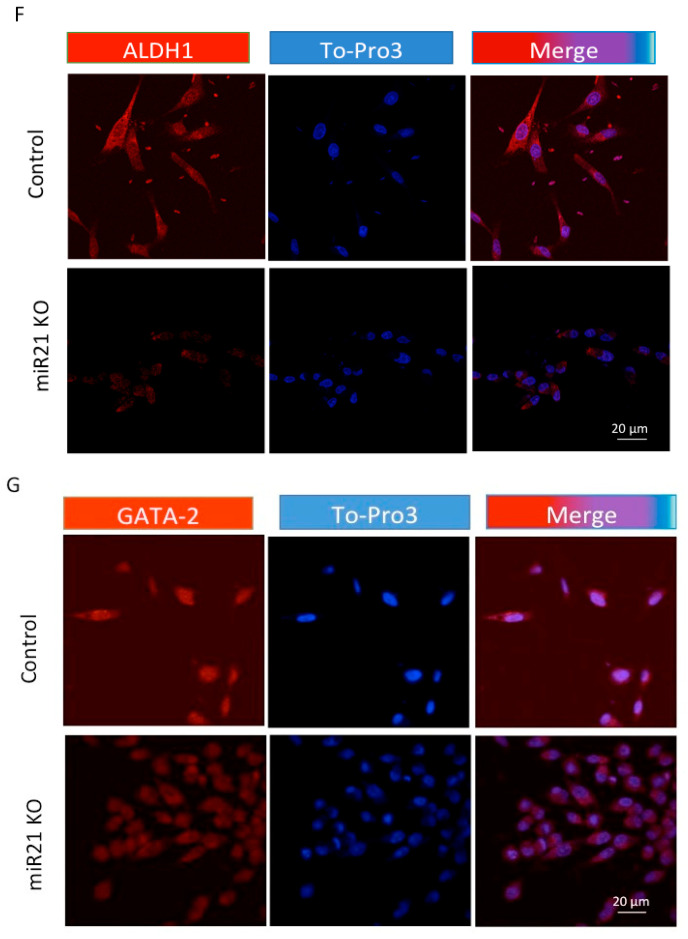
The silencing of miR-21 reduced the mesenchymal phenotype of MDA-MB-231 cells. (**A**) Protein levels of epithelial and mesenchymal markers were tested by Western blotting. β-actin was used as a loading control. The densitometry graph represents protein expression levels of band intensities obtained from at least three independent experiments. The relative band intensities of each sample were calculated by normalizing against β-actin. Each data point represents average ± SD. **** *p* < 0.0001. (*n* = 3) (**B**–**E**). Immunofluorescence assay was performed to show E-cadherin levels and localization, Snail, Zeb-1, and vimentin (green). (**F**–**G**) Cancer stem cell markers ALDH1, GATA2 expressions (red), and localizations were assessed by using immunofluorescence in MDA-MB-231 wt and miR-21 KO cells. ToPro3 (blue) was used for nuclei. KO2 immunofluorescence results were shown as a representative to compare to wt (*n* = 3), scale bar 20 nm.

Since EMT is a key first step for cancer cell invasion and tumor metastasis, the expression levels of EMT markers were assessed both in terms of gene expression and protein levels. The qRT–PCR results showed an increase in E-cadherin mRNA expression in miR-21 KO clones, whereas Snail, vimentin, and Zeb-1 mRNA levels decreased (Figure S3). According to immunoblotting results, ZO-1 and E-cadherin were upregulated compared to control, Snail, vimentin, and ZEB1 were downregulated in miR-21 knockout clone 2 (KO2) and clone 4 (KO4) MDA-MB-231 cells, respectively. Axin, an important scaffold protein of the Wnt signaling pathway, was found to induce functional EMT [60]. However, axin expression levels were not significantly altered (Figure 5A) in KO cells. An essential protein of Wnt signaling, β-catenin, can initiate transcription of EMT-related genes [60,61] the protein levels were decreased in KO cells (Figure 5A). Next, we used immunofluorescence microscopy and observed an increase in E-cadherin and a decrease in Snail, vimentin, and Zeb-1 in miR-21 KO clones when compared to wt (*n* = 3; Figure 5B–E. We then assessed ALDH1 and GATA-2 protein levels using immunofluorescence to assess whether miR-21 was involved in cancer stemness since both ALDH1 and GATA-2 were found to impact cell survival and tumorigenesis in BCa [40,41]. Our results showed that both ALDH1 and GATA-2 protein levels were decreased in the miR-21 KO (*n* = 3; Figure 5F,G).

### 2.4. miR-21 Affects Wnt-11-Mediated Cellular Responses

**Figure 6:**
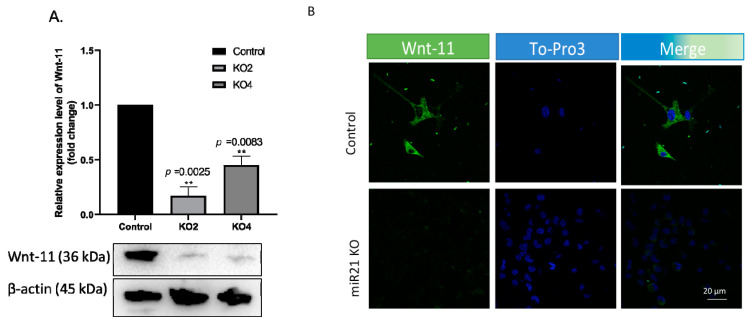
miR-21 deletion downregulated Wnt-11 expression in MDA-MB-231 cells. (**A**) The Wnt-11 expression was examined by Western blot analysis in MDA-MB-231 wt and two selected colonies for *miR-21* KO cells. β-actin was used as a loading control. The densitometry graph represents protein expression levels of band intensities obtained from at least three independent experiments. The relative band intensities of each sample were calculated by normalizing against β-actin. Each data point represents average ± SD. ** *p* < 0.01. (*n* = 3) (**B**) The Wnt-11 expression was examined by immunofluorescence, and ToPro3 (blue) was used for nuclei. Clone 2 (KO2) immunofluorescence results were shown as a representative to compare to wt (*n* = 3), scale bar 20 nm.

We then explored the involvement of miR-21 in the Wnt pathway as the Wnt pathway that has been recognized as a potent regulator of cancer stemness. Our previous data indicated both miR-21 and Wnt-11 expression levels are increased in cancer [22]. We have found that Wnt-11 mRNA and protein levels were diminished in KO clones (Figure 6A; *n* = 3; *p* < 0.05 for all). A similar effect was noted with immunofluorescence analysis in MDA-MB-231 wt and miR-21 KO cells (Figure 6B; *n* = 3).

### 2.5. Regaining miR-21 Reverses the Metastatic Phenotype of MDA-MB-231 Cells

**Figure 7:**
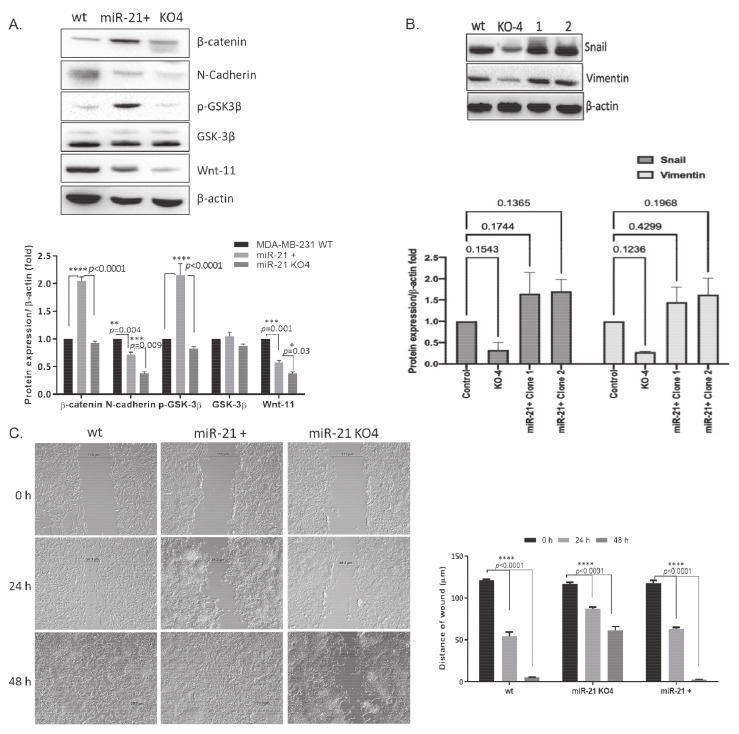
miR-21 increased the motility of *miR-21* KO cells through modulating the Wnt-11, β-catenin, and GSK3β axis to promote mesenchymal markers. (**A**) The total protein was isolated from MDA-MB-231 wt, KO4, and miR-21 retransfected KO4 cells (miR-21+). The expression levels of N-cadherin, vimentin, β-catenin, p-GSK-3β, and Wnt-11 were examined by Western blot analysis. β-actin was used as a loading control. Relative band intensities were calculated by normalizing the values against β-actin. Each data point represents average ± SD. * *p* < 0.05, ** *p* < 0.01, *** *p* < 0.001, **** *p* < 0.0001. (*n* = 3) (**B**). The total protein was isolated from MDA-MB-231 wt, KO4, and miR-21 retransfected KO4 cells (miR-21+). The protein expression levels of Snail and Vimentin were examined by Western blot analysis. β-actin was used as a loading control. Relative band intensities were calculated by normalizing the values against β-actin. Each data point represents average ± SD. **** *p* < 0.0001. (*n* = 3) (**C**). Wound healing assay was performed to compare the motility of KO4 cells with miR-21+ cells. Images were obtained by using light microscopy with 10× magnification. The scale bar is 100 µm. The average distance of wound area was shown following measurement of at least 5 different areas for 0, 24, and 48 h incubation time. Each data point represents average ± SD of three separate experiments.

In order to confirm the potential role of miR-21 in BCa, we transfected miR-21 back to the KO cells, in which six colonies were selected for further studies, and miR-21 expressions were confirmed by qRT–PCR (Figure S4). The Western blot assay was used to confirm the potential effect of miR-21 on the mesenchymal transition of MDA-MB-231 cells (Figure 7A). The expression levels of mesenchymal markers β-catenin, N-cadherin, GSK3β, and p-GSK3β (Ser9), and Wnt-11 were compared within control MDA-MB-231 wt, miR-21+, and miR-21 KO cells. We found that miR-21 + cells were expressing higher levels of selected markers compared to miR-21 KO cells. Transient transfection of miR-21 led to a significant increase of β-catenin and p-GSK-3β levels that were correlated with the upregulation of the EMT mechanism in MDA-MB-231 cells. We found that miR-21 mimic transfected KO BCa cells showed rapid recovery for Snail and vimentin expression levels (Figure 7B). We then tested the cell motility of the miR-21 transfected cells. For that purpose, the wound-healing assay was performed to confirm the potential effect of miR-21 on the migration of MDA-MB-231 cells. miR-21 KO cells reduced the wound healing capability of MDA-MB-231 cells, while both wt MDA-MB-231 and miR-21+ cells aggressively closed the wound site (Figure 7C) within 48 h.

## 3. Discussion

The primary results of this study were as follows: (1) miR-21 expression is a marker of aggressive TNBC. (2) The deletion of miR-21 prevented cell survival and colony formation in MDA-MB-231 cells and reduced EV release and associated EV miR-21 cargo. (3) miR-21 promoted EMT, cellular motility and stem-like markers. (4) miR-21-mediated GSK3 and Wnt-11 signaling, and this determines EMT signaling.

Serum miR-21 has been proposed as a promising cancer biomarker, and reports suggest that compared to CEA and CA153, it offers higher sensitivity for the diagnosis of BCa. In contrast with ER and PR expression levels, serum miR-21 levels in BCa patients was suggested as a promising diagnostic indicator, especially in TNBC patients [62]. In a similar trend, EV miR-21 has been suggested to be a very strong molecular target for the diagnosis of different cancer cases [63]. In the current study, we found that miR-21 EV-cargo levels are significantly reduced in EVs derived from the miR-21 KO BCa cells, compared with EVs derived from control cells. We have previously reported that antiontogenic modulation of EV release is associated with reduced miR-21 content in EVs and that this may affect cellular communication and the microenvironment in cancers [23,24,53,64]. Furthermore, as tumor-derived EVs also carry pro-EMT factors, including TGFβ, caveolin-1, HIF1α and β-catenin [65], our reported findings of reduced EV release in MDA-MB-231 miR-21 KO cells may play some roles in EMT. While the current study is the first to report an EV modulating function for miR-21, using miR-21 KO approaches, the exact pathway(s) of how miR-21 mediates EV biogenesis is still subject to further investigation. Importantly, when assessing EV cargo, we found that EV associated miR-21 expression was 70% reduced from the miR-21 KO cells, compared with control cell EV miR-21 content. Interestingly, it has been shown that EVs derived from MDA-MB-231 tumor cells can modulate mRNA expression of the normal breast cell line MCF10A [48]. Furthermore, EV-miR profiles of the peritoneal lavage fluids from gastric cancer patients showed higher miR-21 expression, which correlated with metastasis [66]. Plasma-EV miR-21 expression levels were also shown to be positively correlated with liver metastasis in colorectal cancer patients [67].

Similarly, previous reports showed that high miR-21 expression levels were associated with increased expression levels of TGF-β1, correlated with advanced tumor grade, negative hormone receptor status, and ductal carcinoma [68]. A recent study reported two additional direct miR-21 targets, programmed cell death 4 (PDCD4) and maspin, a mammary serine protease inhibitor, both of which reduced invasiveness of metastatic BCa MDA-MB-231 cells like TPM1 [69]. The results suggest that, as an oncogenic miRNA, miR-21 has a role in tumor growth and invasion and tumor metastasis by targeting multiple tumor/metastasis suppressor genes. Moreover, it has been shown that the nuclear translocation of β-catenin can regulate the metastasis of gastric cancer [34]. miR-21 could inhibit the expression of PTEN, thus control the downstream Akt signaling pathway to maintain cell survival. Additionally, TPM1, Maspin, MARCKS, and Cdc25A were identified as miRNA-21 target genes [70]. LZTFL1 was also identified as a novel target of miR-21. EV-mediated miR-21 release as a signaling factor is diminished in miR-21 KO BCa cells. miR-21 inhibitors led to drastic alterations in BCa cell proliferation and EMT-mediated metastasis in vitro and in vivo by upregulating LZTFL1. It is proposed that the miR-21/LZTFL1 signaling axis triggers the nuclear translocation of β-catenin to activate the EMT process in BCa cells [34].

Recent studies indicated that miR-21 is involved in the Wnt/β-catenin signaling pathway through its upstream target genes and positive regulation of Wnt; thereby, activating the Wnt/β-catenin signaling pathway and causing β-catenin and cyclin D upregulation. In a similar trend, it was shown that miR-21 expression led to increased LRP6 expression level to activate the WNT/β-catenin signaling pathway [71]. In this study, N-cadherin, E-cadherin, Vimentin, Snail, and Zeb-1, EMT-associated markers were found to be regulated by miR-21. The immunofluorescence results confirmed the qRT–PCR and Western blot results and showed the change in the cellular morphology following miR-21 depletion. The miR-21 expression is significantly reduced, although low residual miR-21 expression still remains. This may reflect a small subpopulation of non-transduced cells that stay in the cell cultures. We noted that the expression level of β-catenin was also miR-21 dependent. Other studies highlighted that, embryonic signals, which are activated during carcinogenesis, which lead to increased mortality rates due to cancer progression. According to the Protein Atlas Database and our previous studies, Wnt-11 is an embryonic marker that is activated during carcinogenesis and is associated with poor prognosis [36]. Furthermore, we previously reported that Wnt-11 leads to neuroendocrine differentiation in prostate cancer, and Wnt-11 regulates proteins including PKC, JNK, NF-κB, Rho, PKA, and PI3K [72,73]. It also has been reported that TGFβ and Wnt signals crosstalk and the TGFβ/BMP4 complex modulate pri-miR-21-processing; thus, mature miR-21 overexpression was found in MDA-MB-468 BCa cells [74]. We have observed that miR-21 regulates Wnt-11 and plays an essential role in cellular migration and colony formation. Cancer stemness marker ALDH1 overexpression was closely linked with the poor prognosis of TNBC. Interestingly, ALDH1 overexpression was found more often in TNBC patients, significantly more than other types of BCa [75]. Moreover, higher expression of ALDH1 in TNBC was linked to chemotherapy resistance [76]. Previously it was reported that suppressing miR-21 sensitizes MCF-7 cells to topotecan, a topoisomerase inhibitor that is in use as a chemotherapeutic agent for BCa [77]. Another study was found that miR-21 overexpression could influence drug response in human non-small cell lung carcinoma [78]. Therefore, miR-21 may act as a chemoresistance factor. miR-21 inhibition led to reduced Bcl-2, an antiapoptotic protein that was linked to drug sensitivity. Although multiple targets of miR-21 have been identified, including PTEN, PDCD4, FasL, SOD3, Cdc25A, RhoB, IL-12p35, Bcl-2, Pellino, TPM1, JAG1 and WNT, knock out strategies for miR-21 expression could lead to upregulation of antiapoptotic family members to promote cell survival sustainability [79]. Similar results were observed for KO-4 cell lines, which displayed a significant upregulation profile for Bcl-xL, but not in Mcl-1 expression levels. Moreover, lentiviral CRISPR/Cas9 vector-mediated miR-21 gene editing has been shown to inhibit cell proliferation, migration, and EMT in ovarian cancer cells; therefore, miR-21 is associated with metastasis and chemoresistance in ovarian cancer [80]. A GATA transcription factor, GATA-2, was found to be involved in EMT processes in prostate cancer and identified as a prognostic marker in colon, hepatocellular, and prostate cancers [81,82,83,84]. It was also found that GATA-2 is a key epigenetic regulator of G9a that associates with cell survival and tumorigenesis of BCa [85]. We have found that GATA-2 expression reduced following miR-21 inhibition. Further studies are needed to understand the possible mechanism of GATA-2 regulation by miR-21 in BCa. miR-21 knockout BCa cells exerted diminished N-cadherin, Snail, and vimentin expression profile, which indicated that miR-21 plays a mechanistic role in EMT progression. miR-21 mimic transfection in miR-21 KO BCa cells Snail and vimentin, but not N-cadherin. Additionally, miR-21 mimic transfection increased wound closure rate compared to miR-21 KO BCa cells. Thus, we concluded that miR-21 mimic transfection increased EMT and increased cell proliferation in BCa cells. It was shown that miR-21 promoted AKT and p-GSK3β inhibitory Ser 9 activation in neural stem/precursor cells [86,87]. In a similar trend, we found that miR-21 mimic transfection increased phosphorylation GSK3β and upregulated β-catenin.

According to previous findings, it was shown that miR21, as an important oncomiR, can promote cell proliferation and migration in MDA-MB-231 cells. In a similar trend with our findings, it was shown that knockdown of miR21 could prevent the cell proliferation in TNBC cells, and it could prevent migration and lung metastasis [88]. Thus, nanoformulations of antagomiR21, which have longer stability, were found promising in the treatment of TNBC cell xenograft animal models [89]. Meta-analysis of 21 relevant studies, which include 2510 TNBC subjects, showed that overexpression miR-155, miR-21, miR-27a/b, miR-374a/b, miR-210, and miR-454 were associated with reduced overall survival ratio in patients. Increased miR-21 expression levels were also predictive of reduced overall survival. Additionally, it was shown that miR-27a/b, miR-210, and miR-454 expression levels were associated with shorter overall survival. miR-454 and miR-374a/b expressions were correlated with disease-free survival status. In our study, we extracted data from the TCGA database and filtered it for TNBC patients [90]. Although miR21 expression levels were positively correlated with reduced overall survival status, there was no significant result for disease-free survival ratio. Therefore, we concluded that the reduced miR21 expression level could be a critical therapeutic target to block EMT progression and prevent the metastatic potential of TNBC cells.

## 4. Materials and Methods

### 4.1. Overall Survival Data

miR-21 related overall survival data from the TCGA Breast cancer BRCA database, consisting of 995 breast cancer patients, were analyzed by using Xena Browser [59].

### 4.2. Cell Culture

MCF-7, SKBR-3, and MDA-MB-231 breast cancer cell lines were obtained from ATCC and cultured in Dulbecco’s modified Eagle’s medium (DMEM) supplemented with 10% FBS (Hyclone, Fisher Scientific, Hemel Hempstead, UK), 100 U/mL penicillin, and 100 μg/mL streptomycin (Invitrogen, Waltham, MA, USA). Cells were maintained at 37 °C in a humidified 5% CO_2_ incubator. (Hera Cell 150i, Thermo, Waltham, MA, USA).

### 4.3. RNA Extraction and qRT–PCR

RNA extraction and qRT–PCR were performed according to our previous studies [22,23]. For the assessment of microRNA, the cells were pelleted for RNA isolation and microRNA analysis. RNA was extracted using Trizol (Sigma, Hertfordshire, UK), and RNA concentration and purity were measured using the NanoDrop Spectrophotometer (Thermo Fisher Scientific, Hemel Hempstead, UK) at 260 nm and 280 nm absorbance. RNAs were reverse-transcribed to cDNA using the qScript microRNA cDNA synthesis kit (Quantabio, Lutterworth, UK) according to the manufacturer’s instructions. The resulting cDNA was used to assess the expression of miR-21, while RNU6 was used as a reference RNA for the normalization of miRNA expression levels. The relative expression of miR-21 was normalized with RNU6 expression levels using the comparative cycle threshold method [4]. The PerfeCTa SYBR Green SuperMix (Quantabio, Lutterworth, UK) was used together with MystiCq microRNA qPCR primers for miR-21 (hsa-miR-21–5p) obtained from Sigma (U.K.). The sequences for U6-snRNA primers were U6 forward, 5′-GCTTCGGCAGCACATATACTAAAAT-3′, and reverse 5′-CGCTTCACGAATTTGCGTGTCAT-3′. The following thermocycling conditions were used: denaturation at 95 °C for 2 min, followed by 40 cycles of 95 °C for 2 s, 60 °C for 15 s, and extension at 72 °C for 15 s. mRNA was isolated using an mRNA extraction kit according to the manufacturer’s instructions (Qiagen, Germantown, MD, USA). cDNA was generated, and the following genes were studied *Wnt-11* [36]; *Vim, Snail, Twist, E-cadherin (CDH1)* as described before [22]. Relative levels of mRNA expression were calculated using the comparative CT/2^−ΔΔCT^ method [91] with *RNA polymerase II (RPII)* as the reference gene for the in-cell-line-based studies. In addition, the standard deviation was calculated as well as a *t*-test using GraphPad Prism 7.00 (La Jolla, CA, USA) software.

### 4.4. CRISPR/Cas9 Assay

The lentiviral CRISPR/Cas9-mediated miR-21 gene editing vectors encoding four different gRNAs, (Table 1), eGFP (control), and Cas9 protein was kindly provided by Dr. Junming Yue, University of Tennessee Health Science Center, USA, and produced as described before [80,92]. Stable cell lines were generated by transducing the MDA-MB-231 cells with the lentiviral CRISPR/Cas9 miR-21 gene editing vectors and selected with 5 μg/mL puromycin.

### 4.5. Genomic Cleavage Assay

To quantify and validate the efficiency of the CRISPR/Cas9 gRNAs, we carried out a genomic cleavage assay using the GeneArt™ genomic cleavage detection kit (Invitrogen, Loughborough, UK). Genomic DNA was extracted from the miR-21 KO and GFP (control) MDA-MB-231 cells, and the genomic cleavage assay was performed following the manufacturer’s protocol. Initial PCR was performed by amplifying the gRNAs targeting region using the primers pri-miR-21F (5′-GGGGATTTCTTGGTTTGTGAA-3′) and pri-miR-21R (5′-ATACAGCTAGAAAAGTCCCTGAAAA-3′) and applying a PCR annealing temperature of 55 °C. 3 μL of each PCR product was run on a 2% agarose gel for amplification checkup, and the remaining amount was used in the cleavage assay. PCR samples were cleaved using a detection enzyme, and cleavage products were run and analyzed on a 4150 TapeStation system using a high sensitivity D1000 ScreenTape and reagents (Agilent, UK). Nondigested samples were included as cleavage control. The total fraction cleaved was represented by two electrophoresis bands/peaks. The sum of the percentage of the integrated area of the two cleaved bands corresponded to the total fraction cleaved. The clone/gRNA presenting the highest cleavage (CRISPR/Cas9) efficiency was selected for further functional studies.

### 4.6. Extracellular Vesicle Isolation

Extracellular vesicles (EVs) were isolated as previously described [23,24] and following the specifications of the International Society for Extracellular Vesicles [93] using stepwise centrifugation as follows: First cells were washed and replaced with FBS free media for 24 h (to remove contaminating EVs), then the supernatant was collected and centrifuged at 4000× *g* for 30 min at 4 °C to remove cell debris and aggregates. Thereafter the supernatant (cell medium containing the EVs) was collected and ultra-centrifuged at 100,000× *g* for 1 h at 4 °C. The EV pellets were resuspended in 1 mL DPBS (sterile filtered through 0.22 μM filter) and ultra-centrifuged again at 100,000× *g* for 1 h at 4 °C, discarding the supernatant. The resulting EV enriched pellets were solubilized in 50 μL DPBS and subjected to nanoparticle tracking analysis (NTA).

### 4.7. Nanoparticle Tracking Analysis (NTA)

Nanoparticle tracking analysis (NTA) for EV profiling was carried out using the NS300 NanoSight (Malvern Panalytical Ltd., Malvern, UK), equipped with an sCMOS camera and a 405 nm diode laser, to enumerate the EVs. Samples (EV enriched pellets, diluted in 50 µL DPBS) were diluted 1:100 in sterile-filtered EV-free DPBS, and the number of particles in the field of view was maintained in the range of 30–50 with a minimum concentration of samples at 5 × 10^7^ particles/mL. Camera settings were according to the manufacturer’s instructions (Malvern Panalytical Ltd.), recording four 90 s videos per sample and averaging the obtained replicate histograms. Each experiment was repeated in three biological replicates.

### 4.8. Hanging Drop Assay

A total of 25 × 10^2^ MDA-MB-231 wt cells and *miR-21* KO cells were seeded drop-by-drop as 10 repeats on the lid of a 60 mm_2_ plate. Briefly, after seeding the cells on a 60 mm plate lid as 10 µL drops, 3 mL 1× PBS was added to the plate for the humidity of the drops. The cells were then incubated at 37 °C in a CO_2_ incubator until 72 h. Spheroid formations were observed under an inverted microscope for 72 h with every 24 h. The diameter of the spheroid size was scaled by using the Olympus Micro DP Manager Image Analysis program for randomly selected at least 10 spheroids. After 72 h, each drop was stained with DiOC6 and examined under fluorescence microscopy (Olympus, Tokyo, Japan).

### 4.9. Colony Formation

Briefly, MDA-MB-231 cells transduced with lentiviral miR-21 gRNA and control vectors were seeded at 1 × 10^4^ density in 6-well plates and incubated for 14 days. After the media was removed and cells were washed with 1× PBS solution, fixed with methanol: acetic acid (3:1) for 20 min at room temperature. After removing the fixing agent, cells were stained with 0.5% *w/v* crystal violet in methanol for 15 min, cells were washed with distilled water, and the morphological images were taken under light microscopy. Image J was used to count the cells for wt, KO2 and KO4.

### 4.10. Western Blot Analysis

MDA-MB-231 wt, miR-21 KO-2, miR-21 KO-4, and miR-21 plasmid-transfected cells were seeded into 60 mm^2^ plates and grown until the cells were 80% confluent. Then, cells were collected after trypsinization with ice-cold PBS. Proteins were obtained by lysis of the cells with an mPER mammalian protein extraction kit (Thermo Fisher Scientific, USA). A Laemmli system with 12% SDS–PAGE gel system was used for the separation of the total protein lysate (total protein/lane was 30–50 μg), which was then transferred onto PVDF membranes through semidry blotting. 5% *w/v* nonfat milk was used to block the membranes overnight at 4 °C. The membranes were washed and incubated with primary antibodies. The following antibodies: Bax, Bcl-xL, Mcl-1, ZO-1, E-cadherin, Snail, vimentin, Zeb-1, β-catenin, Axin-1, GSK-3β, p-GSK-3β (Ser 9), PTEN, and N-cadherin were purchased from Cell Signaling Technology (Danvers, MA, USA). Wnt-11 was purchased from R&D systems (Minneapolis, MN, USA). Membranes were incubated with secondary HRP conjugated anti-rabbit antibodies overnight at 4 °C. Following the addition of lab-made enhanced chemiluminescence solution was prepared with luminol, coumaric acid and Tris-HCl solution (solution A) and Tris-HCl and H_2_O_2_ (solution B), and then the membranes were examined with Chemidoc MP (Bio-Rad, Hercules, CA, USA).

### 4.11. Immunostaining

MDA-MB-231 wt and miR-21 KO cells were seeded into 6-well plates and allowed to settle overnight; the cells were washed with 1× PBS and fixed with 4% formaldehyde for 20 min at room temperature. Cells were washed with 1× PBS twice, then blocked with 5% *w/v* bovine serum albumin (Invitrogen, Loughborough, UK) for 30 min at room temperature. The wells were washed with PBS twice and primary antibodies E-cadherin, Zeb1, Snail (20C8) (Invitrogen, Waltham, MA, USA), vimentin and GSK3B (Cell Signaling Technology; Danvers, MA, USA) ALDH1 (B-5) and GATA2 (H6) Santa Cruz Biotechnology; CA, USA), Wnt-11 (GeneTex; CA, USA) added and incubated for one hour. After washing the cells, either goat anti-rabbit IgG Alexa Fluor or anti-mouse IgG (Thermo Fisher, Oxford, UK) was added and incubated for an hour. Following another washing step with 1× PBS, ribonuclease A 100 mg/mL (Sigma-Poole, Dorset, UK) was added and incubated, gently rocking for 20 min. For counterstaining, 5 µL/mL of 1 nM To-Pro-3 (Thermo Fisher Scientific, Oxford, UK) was dispensed into each well and set gently rocking, then washed twice with PBS for 5 min gently rocking. The results generated were taken from the three biological and technical repetitions. 3–4 mL of 1× PBS were added to each well, Leica TCS SP2 (Leica Microsystems; Milton Keynes, UK) confocal microscope was used to analyze the cells.

### 4.12. Plasmid Transfection for Survival Assay

The pcDNA3-miR-21 plasmid was purchased from Addgene (cat. no.: 21114) and was grown up with ampicillin-contained LB agar. Selected colonies were transferred to the ampicillin LB broth, and then isolated by ZymoPURE plasmid miniprep kit (cat. no.: D4209) (Irvine, USA). miR-21 KO cells were seeded into 6-well plates and miR-21 plasmid transfected to cells by using lipofectamine 2000 (Invitrogen, Waltham, MA, USA) according to the manufacturer’s protocol. Kanamycin was applied by increasing dose until 1000 ng/mL on miR-21 plasmid transfected cells. 6 different colonies were selected for further studies.

### 4.13. Wound Healing Assay

MDA-MB-231 wt, miR-21 KO-4, and miR-21 plasmid-transfected cells were seeded at 80% density cells/well in 6-well plates until cells reached monolayer confluence. A straight scratch on the cell monolayers was created by using a sterile 10 µL pipette tip to obtain a wound in the monolayer. Detached-cells were washed with 1× PBS gently, and cells were then supplemented with a renewed medium containing 5%/*v/v* FBS and incubated at 37 °C. Wound closure was observed every 24 h until the wound closed using a light microscope (Olympus, Japan) (×10). Olympus Micro DP Manager Image Analysis program was used for analyzing the wound area at different time points and presented by bar graphics using GraphPad software (4.04 version).

### 4.14. Determination of ROS Generation

A total of 5 × 10^4^ cells/well MDA-MB-231 wt and miR-21 KO cells were seeded into 6-well plates. After trypsinization and collection of cells into 1.5 µL microfuge tubes, cells were stained with 5 µM DCFH-DA (Molecular probes, Eugene, OR, USA) for 15 min at 37 °C, 5% *v/v* CO_2_. Then stained cells were analyzed by the FL1 filter of BD Accuri C6 flow cytometer.

### 4.15. Statistical Analysis

NTA curves were generated by the NanoSight 3.0 software (Malvern, UK), with the black line representing the mean of the five repetitive readings per individual sample (each treatment group was repeated in three biological replicates) and the red line representing standard error (+/−). Histograms represent the mean of data, and standard deviation (SD) is indicated by the error bars. Significant differences were considered as *p* ≤ 0.05. Statistical analysis such as One-way ANOVA followed by Dunnett’s multiple comparisons test was performed using GraphPad Prism version 8.0.0 for Windows, GraphPad Software, San Diego, California USA, www.graphpad.com.

## 5. Conclusions

In this study, we conclude that miR-21 regulates EMT in BCa. The EMT pathway was controlled by miR-21; this demonstrated the importance of miR-21 as a potential target for the control of cancer stemness. miR-21 expression orchestrates Wnt-11, β-catenin and GSK3β to control the aggressiveness of BCa cells. Further studies are needed to identify related mechanisms in poor prognosis and BCa metastasis in especially TNBC cells; however, our data and others indicate that miR-21 could be a useful target for BCa.

## Figures and Tables

**Table 1:** Sequences of the four guide RNAs (gRNAs) used to knock out the miR-21 gene in the MDA-MB-231 cell line.

gRNA	RNA Sequence
miR-21 gRNA1	CTCATGGCAACACCAGTCGA
miR-21 gRNA2	CTCATGGCAACACCAGTCGA
miR-21 gRNA3	ATGTCAGACAGCCCATCGAC
miR-21 gRNA4	ATGTTGACTGTTGAATCTCA

## Data Availability

The data presented in this study are available in the article and supplementary material.

## Supplementary Materials

Supplementary Materials can be found at https://www.mdpi.com/1422-0067/22/4/1557/s1. Figure S1: Cas9-gRNA editing efficiency could be estimated directly from Sanger electropherograms of short polymerase chain reaction products around the gRNA regions in Cas9-gRNA transduced cells; Figure S2: miR-21 deletion resulted in an increased ROS generation KO2 and KO4; Figure S3: The mRNA expression levels of *E-cadherin*, *Snail, Vimentin*, and *Zeb-1* were examined by qRT–PCR by using WT, KO2, and KO4, RNA polymerase II (RPII) was used as a reference gene (*n* = 6; *p* < 0.005 for all); Figure S4: The miRNA expression levels of control, KO4 and miR21+, were examined by qRT–PCR, U6 was used as a reference gene. Exact *p*-values are indicated, error bars show SD; *n* = 3 biological replicates for all).

## Abbreviations

BCa	Breast cancer
EMT	Epithelial-mesenchymal transition
EV	Extracellular vesicle
miR	microRNA
TNBC	Triple Negative Breast Cancer
TCGA-BRCA	The Cancer Genome Atlas Breast Invasive Carcinoma
ROS	Reactive Oxygen Species
