# Supplementary material for: MiR-21 Is Required for the Epithelial–Mesenchymal Transition in MDA-MB-231 Breast Cancer Cells
Source: Int J Mol Sci. 2021 Feb 4;22(4):1557. doi: 10.3390/ijms22041557 (PMC7913884; doi:10.3390/ijms22041557)
Supplement: Supplementary file 1 [file ijms-22-01557-s001.pdf]

## Supplementary Material

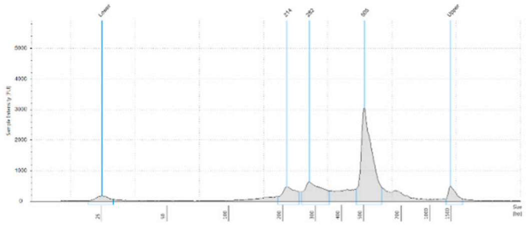

**Sample Table**

| Well | Conc. [pg/µl] | Sample Description | Alert | Observations |
|---|---|---|---|---|
| H1 | 3360 | C+ | ⚠ | Caution! Expired ScreenTape device |

**Peak Table**

| Size [bp] | Calibrated Conc. [pg/µl] | Assigned Conc. [pg/µl] | Peak Molarity [pmol/l] | % Integrated Area | Peak Comment | Observations |
|---|---|---|---|---|---|---|
| 25 | 139 | - | 8560 | - | | Lower Marker |
| 214 | 413 | - | 2970 | 12.29 | | |
| 282 | 729 | - | 3980 | 21.68 | | |
| 505 | 2220 | - | 6760 | 66.02 | | |
| 1500 | 250 | 250 | 256 | - | | Upper Marker |

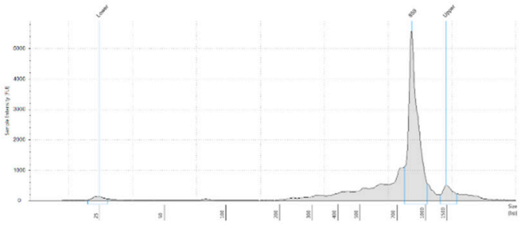

**Sample Table**

| Well | Conc. [pg/µl] | Sample Description | Alert | Observations |
|---|---|---|---|---|
| F1 | 2520 | GFP+ | ⚠ | Caution! Expired ScreenTape device |

**Peak Table**

| Size [bp] | Calibrated Conc. [pg/µl] | Assigned Conc. [pg/µl] | Peak Molarity [pmol/l] | % Integrated Area | Peak Comment | Observations |
|---|---|---|---|---|---|---|
| 25 | 72.7 | - | 4480 | - | | Lower Marker |
| 859 | 2520 | - | 4510 | 100.00 | | |
| 1500 | 250 | 250 | 256 | - | | Upper Marker |

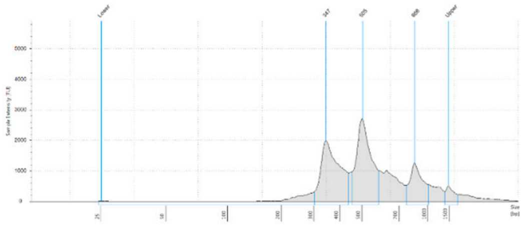

**Sample Table**

| Well | Conc. [pg/µl] | Sample Description | Alert | Observations |
|---|---|---|---|---|
| B1 | 5630 | KO 2+ | ⚠ | Caution! Expired ScreenTape device |

**Peak Table**

| Size [bp] | Calibrated Conc. [pg/µl] | Assigned Conc. [pg/µl] | Peak Molarity [pmol/l] | % Integrated Area | Peak Comment | Observations |
|---|---|---|---|---|---|---|
| 25 | 262 | - | 16100 | - | | edited Lower Marker |
| 347 | 2250 | - | 9970 | 39.94 | | |
| 505 | 2430 | - | 7410 | 43.17 | | |
| 868 | 951 | - | 1690 | 16.89 | | |
| 1500 | 250 | 250 | 256 | - | | Upper Marker |

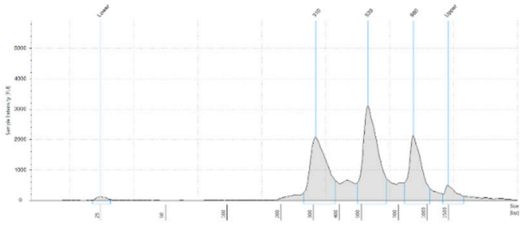

**Sample Table**

| Well | Conc. [pg/µl] | Sample Description | Alert | Observations |
|---|---|---|---|---|
| D1 | 4970 | KO 4+ | ⚠ | Caution! Expired ScreenTape device |

**Peak Table**

| Size [bp] | Calibrated Conc. [pg/µl] | Assigned Conc. [pg/µl] | Peak Molarity [pmol/l] | % Integrated Area | Peak Comment | Observations |
|---|---|---|---|---|---|---|
| 25 | 48.7 | - | 3000 | - | | Lower Marker |
| 310 | 1620 | - | 8060 | 32.67 | | |
| 539 | 2120 | - | 6060 | 42.70 | | |
| 860 | 1220 | - | 2190 | 24.63 | | |
| 1500 | 250 | 250 | 256 | - | | Upper Marker |

**Figure S1.** Cas9-gRNA editing efficiency could be estimated directly from Sanger electropherograms of short polymerase chain reaction products around the gRNA regions in Cas9-gRNA transduced cells.

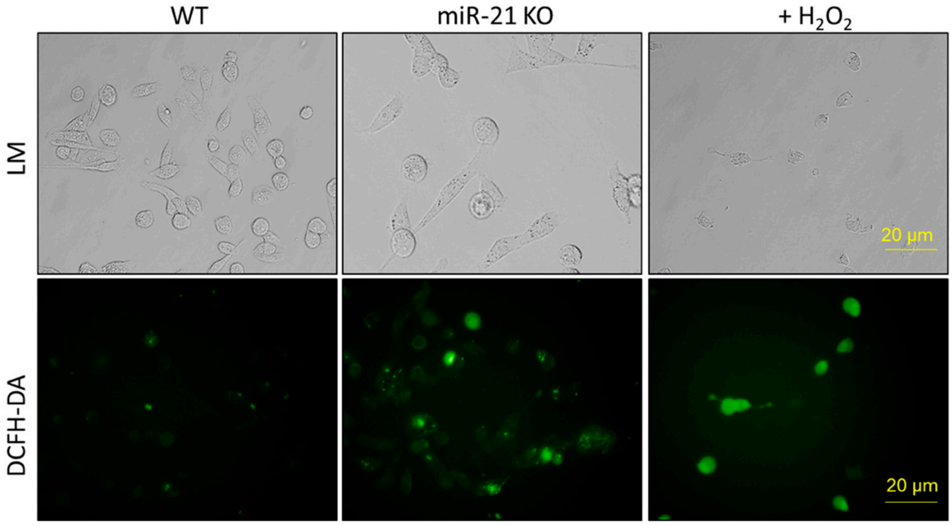


**Figure S2.** miR-21 deletion resulted in an increased ROS generation KO2 and KO4.

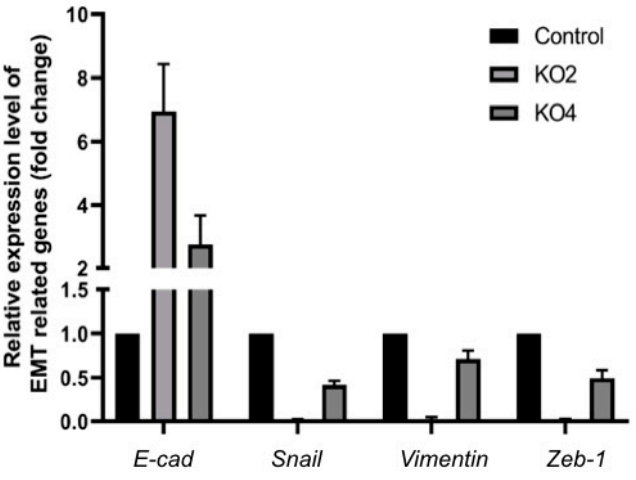


**Figure S3.** The mRNA expression levels of *E-cadherin*, *Snail*, *Vimentin*, and *Zeb-1* were examined by qRT–PCR by using WT, KO2, and KO4, RNA polymerase II (RPII) was used as a reference gene ($n$=6; $p$<0.005 for all).

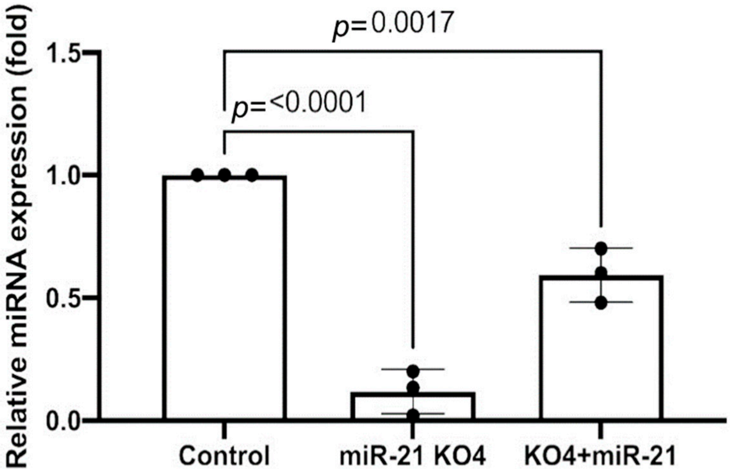


**Figure S4.** The miRNA expression levels of control, KO4 and miR21+, were examined by qRT–PCR, U6 was used as a reference gene. Exact p-values are indicated, error bars show SD; $n$=3 biological replicates for all).
